# Supplementary material for: From sequence to enzyme mechanism using multi-label machine learning
Source: BMC Bioinformatics. 2014 May 19;15:150. doi: 10.1186/1471-2105-15-150 (PMC4229970; doi:10.1186/1471-2105-15-150)
Supplement: Additional file 2 — Java code of ml2db. Additional file ml2db_code.tar.gz contains the Java source code to run the multi-label machine learning experiments and save the results to database. The code’s Javadoc is included. [file 1471-2105-15-150-S2.zip › additional file 2/ml2db/utils/doc/uk/ac/standrews/utils/main/maputils/OneToMany.html]

OneToMany


JavaScript is disabled on your browser.


- Overview
- Package
- Class
- Use
- Tree
- Deprecated
- Index
- Help

- Prev Class
- Next Class

- Frames
- No Frames

- All Classes

- Summary:
- Nested |
- Field |
- Constr |
- Method

- Detail:
- Field |
- Constr |
- Method


uk.ac.standrews.utils.main.maputils

## Class OneToMany<T,U>

- java.lang.Object
- - java.util.AbstractMap<K,V>
  - - java.util.TreeMap<T,U>
    - - uk.ac.standrews.utils.main.maputils.OneToMany<T,U>

- All Implemented Interfaces:
  :   java.io.Serializable, java.lang.Cloneable, java.util.Map<T,U>, java.util.NavigableMap<T,U>, java.util.SortedMap<T,U>

  Direct Known Subclasses:
  :   OneToManyMap

  ---

    

  ```
  public abstract class OneToMany<T,U>
  extends java.util.TreeMap<T,U>
  ```

  See Also:
  :   Serialized Form

- - ### Nested Class Summary

    - ### Nested classes/interfaces inherited from class java.util.AbstractMap

      `java.util.AbstractMap.SimpleEntry<K,V>, java.util.AbstractMap.SimpleImmutableEntry<K,V>`
  - ### Constructor Summary

    Constructors

    Constructor and Description

    `OneToMany()`
  - ### Method Summary

    - ### Methods inherited from class java.util.TreeMap

      `ceilingEntry, ceilingKey, clear, clone, comparator, containsKey, containsValue, descendingKeySet, descendingMap, entrySet, firstEntry, firstKey, floorEntry, floorKey, get, headMap, headMap, higherEntry, higherKey, keySet, lastEntry, lastKey, lowerEntry, lowerKey, navigableKeySet, pollFirstEntry, pollLastEntry, put, putAll, remove, size, subMap, subMap, tailMap, tailMap, values`
    - ### Methods inherited from class java.util.AbstractMap

      `equals, hashCode, isEmpty, toString`
    - ### Methods inherited from class java.lang.Object

      `getClass, notify, notifyAll, wait, wait, wait`
    - ### Methods inherited from interface java.util.Map

      `equals, hashCode, isEmpty`

- - ### Constructor Detail


    - #### OneToMany

      ```
      public OneToMany()
      ```


- Overview
- Package
- Class
- Use
- Tree
- Deprecated
- Index
- Help

- Prev Class
- Next Class

- Frames
- No Frames

- All Classes

- Summary:
- Nested |
- Field |
- Constr |
- Method

- Detail:
- Field |
- Constr |
- Method
